# Gold Mesoporous Silica-Coated Nanoparticles for Quantifying and Qualifying Mesenchymal Stem Cell Distribution; a Proof-of-Concept Study in Large Animals

**DOI:** 10.1021/acsabm.4c01714

**Published:** 2025-02-03

**Authors:** Lotte C. C. Smeets, Ezgi Sengun, Chloe Trayford, Bram van Cranenbroek, Marien I. de Jonge, Katiuscia Dallaglio, Matthias C. Hütten, Mark Schoberer, Daan R. M. G. Ophelders, Tim G. A. M. Wolfs, Renate G. van der Molen, Sabine van Rijt

**Affiliations:** † MERLN Institute for Technology-Inspired Regenerative Medicine, Maastricht University, P.O. Box 616, Maastricht 6200 MD, The Netherlands; ‡ Department of Laboratory Medicine, Laboratory of Medical Immunology, Radboud University Medical Center Nijmegen, Nijmegen 6500 HB, The Netherlands; § Department of Pediatrics, Maastricht University Medical Center+, MosaKids Children’s Hospital, Maastricht 6200 MD, The Netherlands; ∥ GROW Research Institute for Oncology and Reproduction, Maastricht University, Maastricht 6200 MD, The Netherlands; ⊥ Global Rare Diseases R&D, Chiesi Farmaceutici S.p.A., Parma 43122, Italy; # Division of Neonatology, Department of Pediatrics, University Hospital RWTH Aachen, Aachen 52074, Germany

**Keywords:** mesenchymal stem cells, cell tracking, mesoporous silica nanoparticles, immunomodulation, large animal model

## Abstract

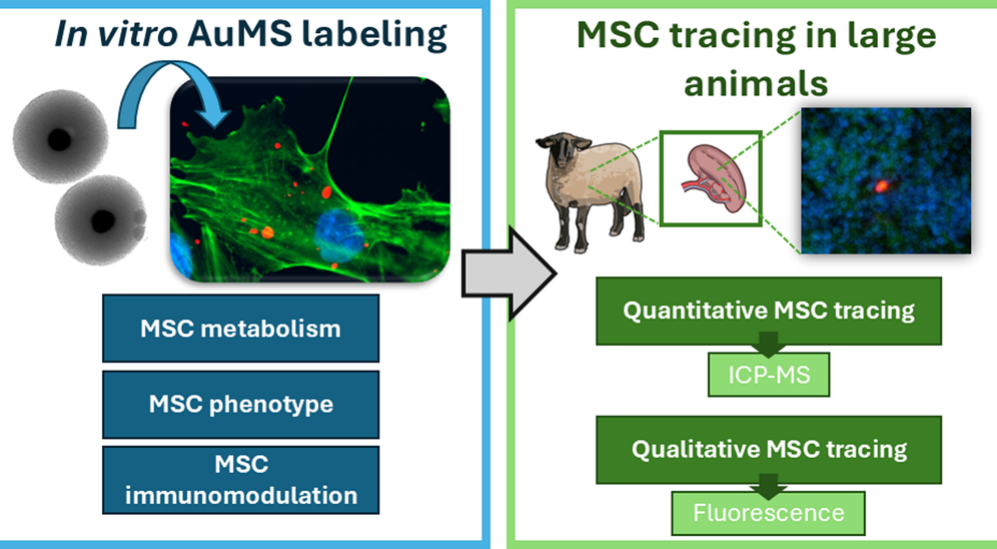

Mesenchymal stem cells (MSCs) have demonstrated promising therapeutic potential across a wide range of diseases including (multi) organ injury in neonates. Despite the reported preclinical successes of MSC therapy, a major challenge in their clinical translation is a limited understanding of their biodistribution after administration. This knowledge gap needs to be addressed to allow clinical implementation. Accordingly, in this study, we propose that silica-coated gold nanoparticles (AuMS) are a promising tool for in vivo MSC tracing. This study explores the use of AuMS for both qualitative and quantitative MSC tracking in vivo after intravenous (I.V.) administration in a translational ovine model of preterm birth. Additionally, we assess the impact of AuMS labeling on the immunomodulatory functions of MSC, which play an important role in the therapeutic potency of these cells. Quantitative and qualitative assessment of AuMS-labeled MSC was performed in vivo using fluorescent microscopy and inductively coupled plasma mass spectrometry (ICP–MS), respectively. AuMS localization in the liver, spleen, and lung was demonstrated. In vitro studies showed that AuMS cellular uptake occurs within 6 h and remains internalized up to 72 h. Labeled MSC maintained their immune phenotype and did not alter their immunomodulatory capacity and proliferation abilities. Overall, we demonstrate that AuMS is a promising, biocompatible nanoprobe for MSC tracing up to 72 h post-I.V. administration.

## 1 Introduction

Mesenchymal stem cells (MSC) have attracted significant attention in regenerative medicine due to their ability to differentiate into many cell types such as osteoblasts, neuronal cells, adipocytes, and myocytes.^1^ In addition to their differentiation potential, MSC exert immunomodulatory capabilities in which interactions with regulatory T cells (Treg) and monocytes play an essential role.^1−3^ MSC immunomodulatory potential is mainly exerted via direct interactions with immune cells and the secretion of multifunctional molecules. These unique immunomodulatory functions make MSCs invaluable for tissue regeneration caused by inflammatory diseases.^1^

Preterm birth, defined as birth before 37 weeks of gestational age, is a significant contributor to neonatal mortality and morbidity.^4^ Intrauterine inflammation is a leading risk factor for preterm birth and can affect almost all organ systems of the developing fetus.^4−6^ Therefore, neonates are at high risk for developing complications of prematurity including necrotizing enterocolitis, bronchopulmonary dysplasia, and cerebral palsy resulting in life-long consequences.^7^ Since neonates face developmental delays and due to the importance of inflammation in the pathophysiology of neonatal diseases, MSC-based therapies have high therapeutic potential because of their immunomodulatory and regenerative capabilities.^5,8^

Preclinical research showed that MSCs protect the preterm brain after hypoxia-ischemia by modulating the peripheral T-cell response.^9^ Moreover, it is demonstrated that MSC therapy prevents arrested alveolar growth.^10^ Although MSCs are promising for treating complications of preterm birth in preclinical research, MSC-based therapies face challenges in their clinical translation.^11^ A major challenge remains the limited understanding of how MSCs are distributed in the body and the sustainability of response post-transplantation.^12−15^ Tracing methods that can quantitatively and qualitatively monitor cell biodistribution, fate, and mode of action post-transplantation are urgently needed in order to guide a more successful clinical translation of cell-based therapies.^16,17^

Within the field of MSC transplants, multiple techniques are applied to label MSC including bromodeoxyuridine, green fluorescent protein, fluorescent dye, lac-Z reporter, and Y chromosome marker.^18^ However, they can be cytotoxic and most of these techniques rely on fluorescent labels which are not optimal since they lose their intensity over time making long-term quantitative analysis of stem cells impossible.^18^ Moreover, due to high autofluorescence and the sensitivity of the imaging techniques required to detect low concentrations of labeled cells, it is impossible to identify individual cells in large animal models using fluorescent probes. Isotope labeling and magnetic resonance imaging (MRI) are other noninvasive techniques. However, cell tracing using MRI can lead to false positive results caused by the release of contrast media from dead cells. Besides, MRI cannot detect proliferated and differentiated cells since contrast media cannot be distributed to dividing cells.^18,19^ Isotope labeling methods applied in single photon emission computed tomography (SPECT) are used limitedly due to the restricted availability of specific MSC markers needed for cell-specific tracing.^18^ Another challenge of MSC tracing in preclinical studies is the detection limit of the tracing techniques.^20^ This represents a major challenge in cell tracing since tracing techniques are unable to detect cell migration in low concentrations, for example, to off-target organs, due to their low sensitivity.^21^ Subsequently, this leads to substantial variations in the reported pharmacokinetics and provides an incomplete understanding of cell distribution, which is essential for translating cell-based therapies into the clinic. Hence, cell tracing techniques must be highly sensitive to be able to detect labeled cells in large animal models and should not affect MSC function, morphology, and differentiation capacity.^22^

Nanoparticles (NP) are considered promising in vitro and in vivo cell tracing tools due to their unique optical and material properties. Specifically, their nanoscale dimension (diameter ranging between 1 and 100 nm) enables them to be easily transported across cell membranes and reach cell organelles.^17,23^ Their high surface area-to-volume ratio enables an enhanced interaction with cellular components.^23^ Many types of NP have been developed for a large variety of bioimaging purposes including luminescent (upconversion) NP for the detection of early stage breast cancer,^24^ cy7.5 silica NP for monitoring drug diffusion,^25^ iron oxide NP-micelles for thrombosis monitoring,^26^ treatment, and characterization, and gold NP for the determination of proteases in tumor-bearing mice.^27,28^

Gold (Au)-based NP (AuNP) are inorganic noble metal NP with unique physical and chemical properties including conductivity, surface plasmon properties, biocompatibility, and X-ray attenuation, that make them ideal for bioimaging applications.^29,30^ Hence, multiple techniques can be used to image AuNP including computed tomography (CT), X-ray imaging, surface-enhanced Raman spectroscopy, optical coherence tomography, and photoacoustic imaging.^30^ In addition, elemental analysis could be performed with inductively coupled plasma mass spectrometry (ICP–MS), a sensitive, accurate, and precise technique. This technique enables elemental analysis in trace and ultratrace concentration ranges and could,^31^ therefore, be an excellent method for quantifying Au as a measurement for cell distribution in vivo.

In order to use NP such as AuNP for stem cell tracing applications, the effect of NP labeling on MSC needs to be understood. Functional deficits of MSC caused by NP labeling can lead to alterations in migration profile and function including their prominent immunomodulatory capabilities.^32,33^ Hence, careful monitoring of NP-labeled MSC cellular functions is a prerequisite for assessing biodistribution in (pre)clinical studies. For example, a study by Nold et al. (2017) showed that MSC exposure to AuNP can lead to alterations in migration and proliferation behavior.^33^ Another study showed that AuNP-labeled MSC maintained its immunophenotype based on surface expression markers.^33^ In addition to cellular functions, it is of significant importance to assess MSC distribution over time. Prior studies indicated that AuNP-labeled MSC could be traced for up to 21 days using CT imaging and fluorescent probes loaded onto the AuNP in small animal models.^34,35^

Although AuNP has promising optical properties for cell labeling, they normally require surface coating with proteins, polymers, or other materials to increase their biocompatibility, functionality, and cellular uptake. For example, mesoporous silica (MS) surface coating has been used to enhance their stability and biocompatibility. Moreover, MS coating can be used to expand their imaging capabilities by the incorporation of fluorescent probes within the coating to create multimodal imaging tools.^30,36^ This is advantageous as multimodal imaging allows us to combine imaging modalities which expands and improves the scope of available information. In addition, the limitations of single imaging can be overcome by synergetic image reconstruction.^30,37,38^

In this study, we developed MS-coated AuNP (AuMS) as a multimodal in vivo cell tracing tool. AuMS’ design provides the opportunity to quantify cell distribution based on their gold core by using ICP–MS and the MS coat enhances biocompatibility and contain stably conjugated fluorescent probes to allow fluorescent imaging on tissue slices. Further, we assessed the impact of AuMS on the ability of MSC to modulate immune cell behavior. In vitro, we determined AuMS cytotoxicity and internalization in MSC, the effect of AuMS labeling on immune function, and the phenotype of MSC. As proof of concept, AuMS labeled MSC (AuMS-MSC) biodistribution was determined quantitatively and qualitatively in a preclinical ovine model of preterm birth. This preterm ovine model is well established for the investigation of preterm birth and valuable to study the distribution and key functions of MSCs as the model closely mimics the clinical conditions of preterm-born babies.^39^ This study is the first to show that AuMS can be used as a promising cell tracing tool to qualitatively and quantitatively assess MSC distribution in large animal studies.

## 2 Methods

### 2.1 Study Approval

Animal experiments were approved by the Dutch Central Authority for Scientific Procedures on Animals (AVD107002022869) and the Animal Welfare Body of Maastricht University (PV2022-001).

All volunteers provided written informed consent. Ethical approval for using healthy blood controls was obtained from the ErasmusMC Medical Ethics Committee (METC) under project number NL40331.078..

### 2.2 AuMS Synthesis and Characterization

AuMS of 60 nm were synthesized according to previously reported methods by Trayford et al. (2022).^30^ Briefly, citric acid (3 mL, Sigma-Aldrich) was added to chloroauric acid (97 mL, 0.3 mM, VWR) under reflux at 100 °C to synthesize 22 nm size seeds. The reaction continued until a deep red color was achieved. Before growing 60 nm AuNP, the seed number was determined by UV–vis spectrometry. Seed concentration was calculated by using the following formula  (*A* = absorbance at 450; ε = extinction coefficient of 18 nm AuNP (ε450 = 3.87 × 10^8^)). Next, a total number of 9.5 × 10^11^, 22 nm seeds were added to chloroauric acid (2.5 × 10^–5^ M) and rapidly stirred. Subsequently, hydroquinone (454 μL, 50 mM, Merck) and citric acid (50 mM, 680 μL) were added, and the reaction was continued for 1 h. Cetrimonium bromide (CTAB) (1 mL, 0.1 M, Fisher Scientific) was added to the reaction and stirred overnight at room temperature to change the surface ligand. Next, excessive amounts of CTAB were removed from the nanoparticle solution by collecting the particles with centrifugation, washing, and redispersing in H_2_O (100 mL). Subsequently, the size of the synthesized CTAB-stabilized AuNP was determined by dynamic light scattering (Malvern Panalytical) and UV–vis spectrometry by using the same calculation method as described earlier [ (ε450 = 1.73 × 10^10^)].

The synthesis was continued by coating the 60 nm AuNP with mesoporous silica. For this, 6.5 × 10^–6^ mol of CTAB stabilized AuNP were redispersed in H_2_O and sonicated (Bransonic, Thermo Fisher Scientific). Next, CTAB (0.273 g; 7.5 × 10^–4^ mol) was dissolved in 170 mL H_2_O and 75 mL absolute ethanol and stirred at 35 °C. After the solution was transparent, NH_3_ (100 μL, 25% vol, Carl Roth) was added and the solution was stirred for a further 5 min. Next, the concentrated redispersed AuNP were added to the solution. 3-Mercaptopropyl trimethoxysilane (MPTES) (5 μL, 5.3 μmol, Sigma-Aldrich) and tetraethyl orthosilicate (TEOS) (60 μL, 60.6 μmol, Sigma-Aldrich) were added dropwise to the reaction, and the temperature was increased to 60 °C. After 5 min, the second addition of silica sources was introduced by a dropwise administration of TEOS (74 μL, Sigma-Aldrich) and rhodamine B isothiocyanate (RITC)-3-aminopropyl triethoxysilane (APTES) (72.5 μL) solution. RITC-APTES was prepared 24 h before starting the coating reaction in the dark by adding RITC (5 mg, bioconnect) to 44 μL of APTES (molar ratio 1:10) in 1 mL absolute ethanol. After 30 min, the third addition of silica sources was introduced by adding dropwise APTES (15 μL, 15.8 μmol) and TEOS (5 μL, 5.4 μmol) and the reaction was stirred overnight. The particles were collected and heated under reflux (90 °C) in 100 mL absolute ethanol containing NH_4_NO_3_ (2 g, Sigma-Aldrich) for 45 min to perform template extraction. Next, the particles were collected and washed twice with ethanol. Lastly, to remove CTAB from the mesoporous, the particles were washed with 1 M HCl. The synthesized AuMS were stored in absolute ethanol at −20 °C until use.

### 2.3 Peripheral Blood Mononuclear Cell Isolation and Negative Selection of CD4^+^ T Cells and CD14^+^ Monocytes

Blood samples for peripheral blood mononuclear cells (PBMC) isolation were collected in EDTA tubes (BD, Labware). Blood was obtained from healthy individuals and prior to blood sampling, donors signed a written informed consent for scientific use according to Dutch law. PBMC were isolated using AutoMACS (Miltenyi Biotec GmbH). CD4^+^ T cells and CD14^+^ monocytes were isolated by negative selection using either CD4^+^ T cell Isolation Kit (Miltenyi) or Pan Monocyte Isolation Kit (Miltenyi), respectively.

### 2.4 MSC Culturing and AuMS Labeling

Human umbilical cord (HUC)-MSC were provided by Chiesi Farmaceutici S.p.A, Italy. MSC were cultured in complete growth medium which contains 2000 U/L heparin (Leo Pharma B.V) and 5% human platelet lysate (provided by Chiesi Farmaceutici S.p.A, Italy). MSC were thawed at 37 °C for 4–6 min, centrifuged at 500xg for 10 min at room temperature, and the pellet was dissolved in 15 mL complete MSCs growth medium. After trypan blue counting, cells were seeded in either 96, 24, or 6 well plates for in vitro experiments and T75 culture flasks for in vivo experiments (Greiner Bio-One).

For generating AuMS labeled MSC (AuMS-MSC), 10.000 and 50.000 MSC were seeded either to 96- or 24-well plates for MTT assay, CD4^+^ T and CD14^+^ monocyte coculture experiments and live/dead staining, and AuMS internalization experiments, respectively. For ovine experiments, 1.25 × 10^6^ MSC were seeded in T75 culture flasks. After seeding, MSC were incubated at 37 °C and 5% CO_2_ for 24 h. After attachment, the cell culture medium was slowly removed and replaced with 50 μg/mL AuMS-containing medium and incubated at 37 °C and 5% CO_2_ for 24 h. Before the coculture experiments, AuMS-MSC were washed once with PBS.

### 2.5 Cell Stimulation and AuMS-MSC Coculture

100.000 CD4^+^ T cells were seeded in 96 well U-bottom plates (Greiner Bio-One) with complete RPMI medium (Thermo Fisher Scientific), supplemented with 10% human processed serum (HPS) (manufactured in-house), 1 mM pyruvate, 2 mM glutamax, 100 U/mL penicillin, and 100 μg/mL streptomycin (all Thermo Fisher Scientific). CD4^+^ T cells were activated with αCD3/CD28 beads (Dynabeads Human T-Activator, GibcoThermo Fisher Scientific) in 1:5 (bead-to-cell) ratio for 24 h. After 24 h, activated or control CD4^+^ T cells were resuspended and transferred to either MSC or AuMS-MSC in a 1:5 (MSC/CD4^+^) cell ratio. Cells were cocultured for 5 days, and immunophenotyped after 24 and 120 h.

500.000 CD14^+^ monocytes were seeded in 24 well flat bottom plates (Nunc cell culture plates, Thermo Fisher Scientific) with complete RPMI medium (Thermo Fisher Scientific). CD14^+^ cells were stimulated with 10 ng/mL lipopolysaccharide (LPS) for 24 h. Subsequently, cells were resuspended and transferred to either MSC or AuMS-MSC with 1:5 (MSC/CD14^+^) ratio. Cells were cocultured for up to 3 days, and flow cytometry measurements were acquired every other day.

### 2.6 Live–Dead MSC Staining

50.000 MSCs were seeded in a 24-well plate and incubated at 37 °C and 5% CO_2_ for 24 h. Twenty-four h after seeding, MSC culture medium was removed and replaced with 50 μg/mL AuMS-containing culture medium. MSCs were incubated with AuMS-containing culture medium for 6, 24, and 48 h. Immediately after incubation, AuMS-containing culture medium was removed, replaced with calcein AM (1:1000, Invitrogen by ThermoFisher Scientific) containing culture medium, and incubated for 30 min in the dark at room temperature to stain nonfixed MSCs for live cells. Calcein AM containing culture medium was removed, replaced with red dead cell stain kit (1:1000 Invitrogen by ThermoFisher Scientific) containing medium, and incubated for 30 min in the dark at room temperature. Next, cells were washed twice with PBS and imaged using an automated inverted Nikon TI-E fluorescence microscope 20× objective.

### 2.7 Flow Cytometry

#### 2.7.1 Different Staining Strategies Were Used for CD4^+^ T, MSC, and CD14^+^ Monocyte Experiments

For CD4^+^ T cells, samples were transferred to 96 well V-bottom plates (Greiner Bio-One) and washed once with flow cytometry buffer (FACS Buffer containing 0.2% bovine albumin, BSA (Sigma-Aldrich), in PBS (Fresenius Kabi)). Samples were stained with surface markers for 20 min at room temperature, following viability staining (30 min at 4 °C). After washing the samples twice with FACS Buffer, cells were fixed and permeabilized according to the manufacturer’s instructions (eBioscience), then cells were stained intracellularly, as previously described.^40^ Samples were measured using Navios flow cytometry (Beckman–Coulter).

For CD14^+^ monocytes and MSC staining, the procedure was performed on ice to prevent cell attachment to the plate. After washing the cells with FACS buffer, cells were stained with viability dye for 30 min at 4 °C. Stained samples were washed once with FACS buffer. Then cells were stained with surface marker mix for 30 min on ice in the dark. Monocyte samples were washed twice with FACS buffer before measurement. MSC samples were further permeabilized and fixed according to the manufacturer’s instructions (eBioscience). After 30 min of intracellular staining at 4 °C in the dark, cells were washed twice with permeabilization buffer. Finally, samples were resuspended in FACS buffer. Both CD14^+^ monocytes and MSC were acquired in Sony ID7000 spectral cell analyzer (Sony Biotechnology).

Table S1 summarizes the antibodies used for flow cytometry. Flow cytometry data were analyzed using Kaluza Software (Beckman Coulter, analysis version 2.1). Graphics and statistical tests were performed in R version 3.6.2. Differences between the levels of outcome were explored using paired *t*-test with mean average. The level of statistical significance was set at *p < 0.05, **p < 0.01, ***p < 0.001, ****p < 0.00001.

### 2.8 MTT Assay

To evaluate the metabolic activity of AuMS-MSC, 10.000 cells were seeded per 96-well-flat-bottomed plate (Greiner Bio-One) in 150 μL complete MSC growth medium. Cells were incubated overnight at 37 °C and 5% CO_2_. After 24 h incubation, the plates were centrifuged at 250*g* for 2.30 min. The supernatant was removed. MSC were treated with either 0, 10, 50, 250, or 500 μg/mL AuMS. As a negative control, MSC growth medium was used. As a positive control, MSCs were treated with beta-propiolactone to kill the cells. Twenty-four h later, 100 μL fresh culture medium with 10 μL of 12 mM 2-(4,5-dimethylthiazol-2-yl)-2,5 dietrazolium bromide (MTT) (Thermo Fisher Scientific) was added to each well. The cells were incubated at 37 °C and 5% CO_2_ for 4 h. Subsequently, cells were centrifuged again at 250*g* for 2.30 min, medium was removed. 50 μL DMSO was added to each well. Plates were incubated at 37 °C for 10 min. Absorbance was measured at 540 nm at TECAN Infinite F50 (TECAN).^40^ The level of statistical significance was set at *p < 0.05, **p < 0.01, ***p < 0.001, ****p < 0.00001, by using a *t*-test with mean average.

### 2.9 In Vitro AuMS-MSC Internalization and Localization Using Fluorescence Microscopy and ICP–MS

Fluorescent microscopy was performed to assess the internalization and localization of AuMS in MSC. 2.56 × 10^5^ MSC were seeded on coverslips in 6 well plates. Cells were exposed to 50 μg/mL AuMS for 6, 24, 48, and 72 h and fixed using 4% paraformaldehyde. Next, cells were washed twice with PBS and permeabilized with 0.1% Triton-x (Merck) for 10 min. Cell nuclei were stained with DAPI (1 μg/mL; Thermo Fisher Scientific), and actin filaments were stained using phalloidin-Alexa fluor 488 for 20 min (1/150; Sigma-Aldrich). Coverslips were mounted on microscope glass slides using fluorescence mounting medium (Dako Omnis). Finally, samples were analyzed using an automated inverted Nikon TI-E fluorescence microscope 60× oil objective.

ICP–MS was performed to quantify AuMS uptake in MSC. MSC pellets were diluted in aqua regia [HCl 30% (VWR) and HNO_3_ 69% (ROTIPURANUltra 69%, Carl Roth) (4:1)]. Accordingly, diluted samples were sonicated in an ultrasonic bath (Bransonic, Thermo Fisher Scientific) at 35 °C overnight and homogenized by microwaving (3 × 30 s). Next, homogenized samples were diluted (1:10) in freshly prepared matrix solution (1% HCl and 20 ppb ruthenium (VWR) as an internal standard). A gold standard curve ranging from 4.89 ng/L to 2.5 μg/L was prepared by diluting gold 100 ppb (VWR) in the matrix solution. In addition, a cell standard curve of labeled AuMS-MSC was prepared to be able to quantify the number of MSC based on gold concentrations (ng/l). ICP–MS was performed using iCAP RQ ICP–MS (Thermo Fisher Scientific).

### 2.10 In Vivo Quantitative and Qualitative Tracing of MSC Using ICP–MS and Fluorescence Microscopy

AuMS-MSC were I.V. administered to lambs to assess the eligibility of AuMS as nanoprobes for MSC tracing in large animals. Briefly, time-mated fetuses of Texel ewes were intramuscularly treated with betamethasone (Celestone Chronodose, 11.4 mg) to stimulate fetal lung maturation at 132 days of pregnancy. At 133 days of gestational age (term ∼150 days), lambs were delivered through a Cesarean section with a modified EXIT procedure including primary intubation and catheter placement as described earlier.^41^ Subsequently, lambs were treated at the lamb intensive care unit mimicking the neonatal intensive care unit conditions including mechanical ventilation for 12 or 72 h.^42^ Immediately after birth, lambs received an I.V. dose of 20 × 10^6^ AuMS-labeled MSC/kg bodyweight via a separate peripheral venous access. Twelve h or 72 h post-AuMS-MSC administration, fetuses were humanely killed with an overdose of pentobarbital, and organs were collected and snap-frozen for analysis.

ICP–MS was performed to quantitatively assess gold content in the liver, lung, and spleen 12 h post I.V. AuMS-MSC administration. Snap-frozen tissue samples were weighed in Eppendorf tubes. Next, samples were freeze-dried overnight, reweighed, and transferred to graduated glass vials (VWR). Subsequently, tissue samples were dissolved in freshly prepared aqua regia solution (30% ICP-grade HCl (VWR) and 69% ICP-grade HNO_3_ (ROTIPURANUltra, Carl Roth) (4:1); 1 mg/50 μL and sonicated in an ultrasonic bath (Bransonic, Thermo Fisher Scientific) at 35 °C overnight. After dissolving the tissues, samples were homogenized by microwaving (3 × 30 s, 600 W). Homogenized samples were diluted (1:10) in freshly prepared matrix containing 1% HCl and 20 ppb ruthenium (VWR) as an internal standard. For the quantification of the gold concentration in the samples, a gold standard curve was prepared ranging from 2.5 μg/L to 4.89 ng/L by diluting gold stock solution (VWR) in matrix. Moreover, a cell standard curve was prepared from the AuMS-MSC administered to the lamb to be able to quantify the number of MSCs per tissue based on gold concentration (ng/L). Lastly, samples were measured using iCAP RQ ICP–MS (Thermo Fisher Scientific).

Fluorescent microscopy was used to assess the presence of AuMS-MSC in lamb liver, lung, and spleen. And to assess whether MSC are localizing with AuMS 72 h postadministration. A cryotome (LEICA CM3050 S) was used to cut 10 μm thick cryosections. Sections were permeabilized using 0.1% Triton-x for 10 min at room temperature. For blockage of nonspecific binding, sections were washed thrice using PBS and treated with 0.1% bovine serum albumin for 30 min. Next, sections were stained with human nuclear antigen–antibody (1:100; Abcam) at 4 °C overnight. Subsequently, sections were washed with PBS and stained against human nuclear antigen–antibody using goat-antimouse Fluor 647 (1:100, Thermo Fisher Scientific) for 1 h at room temperature. Sections were using PBS and stained against actin using phalloidin-Alexa Fluor 488 (1:500; Thermo Fisher), and DNA using DAPI (1 μg/mL, Sigma-Aldrich) for 20 min at room temperature. Lastly, sections were washed with PBS and mounted with DAKO fluorescent mounting medium. Samples were analyzed using an automated inverted Nikon TI-E fluorescence microscope 40× objective.

## 3 Results

### 3.1 Synthesis and Characterization of AuMS

**Figure 1:**
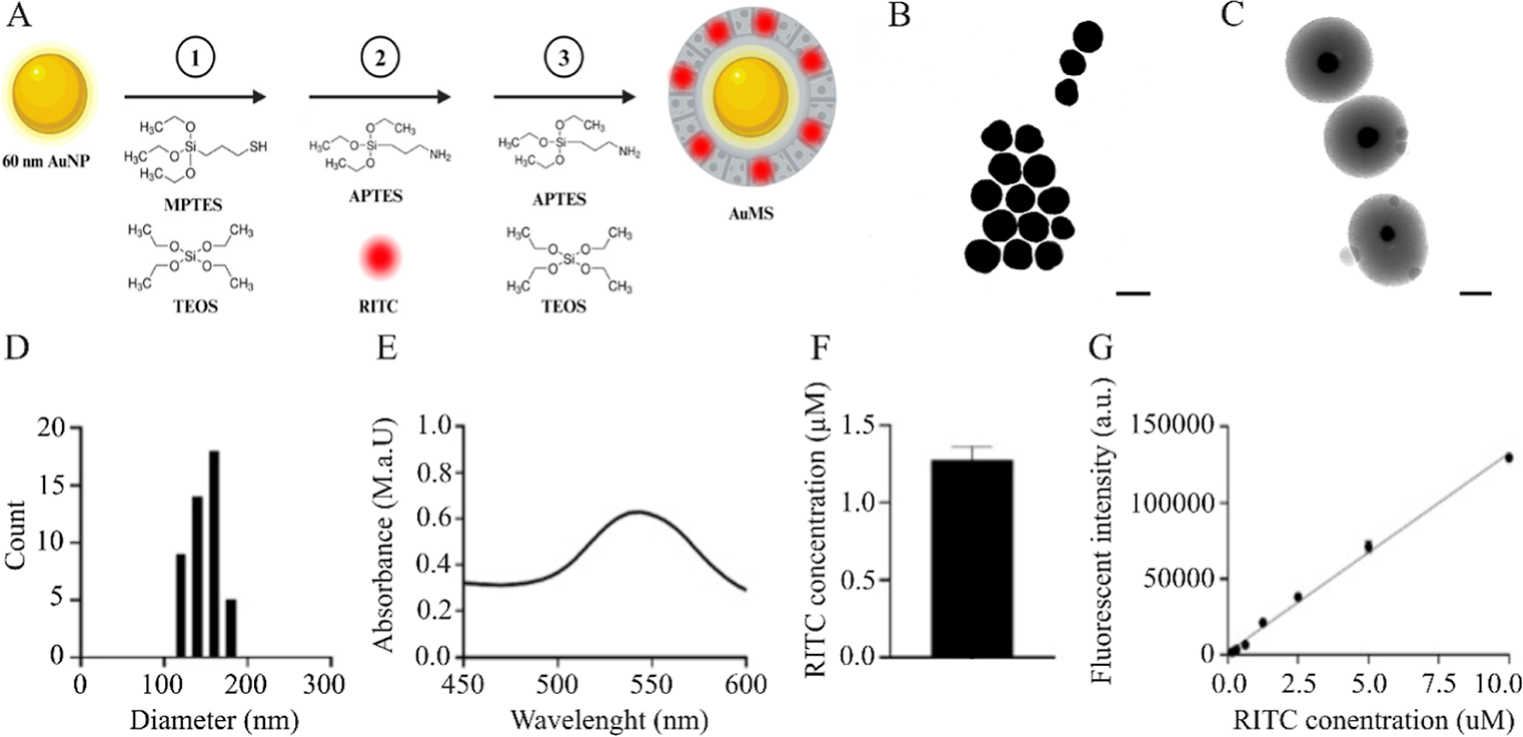
Synthesis and characterization of AuMS. (A) MTES, TEOS-APTES, and APTES were respectively added to CTAB stabilized 60 nm AuNP to synthesize AuMS containing a thiol core amine surface-functionalized rhodamine-B mesoporous silica surface layer (Created with Biorender.com). (B) TEM image of 60 nm AuNP (scale bar = 50 nm) (C) TEM image of AuMS (scale bar = 100 nm). (D) AuMS size analysis of 30 particles (E) AuMS fluorescence absorption analysis by UV–vis spectrometry (F–G) RITC concentration (μM) and fluorescence intensity in the AuMS using a RITC standard curve.

AuNP with a silica coat (AuMS), containing a thiol-functionalized core and amine-functionalized surface were successfully synthesized (Figure 1). To achieve this, 60 nm AuNP were retrieved from growing 20 nm seeds using an adapted hydroquinone-mediated reduction method. Afterward, the AuNP was coated with mesoporous silica using a modified Stöber process (Figure 1A)^30^ to enhance AuNP’s biocompatibility. Transmission electron microscope (TEM) images confirmed the successful synthesis of 60 nm AuNP (Figure 1B), and the round shape and uniform mesoporous structure and size of our synthesized AuMS (Figure 1C,D). AuMS optical properties were measured using UV–vis spectroscopy (Figure 1E). AuMS’ fluorescent properties were achieved by incorporating RITC in the mesoporous silica coating (Figure 1A), allowing visualization of the AuMS with fluorescent microscopy (Figure 1F, G). Here we demonstrated successful synthesis of AuMS containing a RITC-doped MS coat functionalized with −SH groups at the AuMS core and NH_2_ at the surface.

### 3.2 AuMS are Internalized in the MSC Cytoplasm 6 h after Exposure

**Figure 2:**
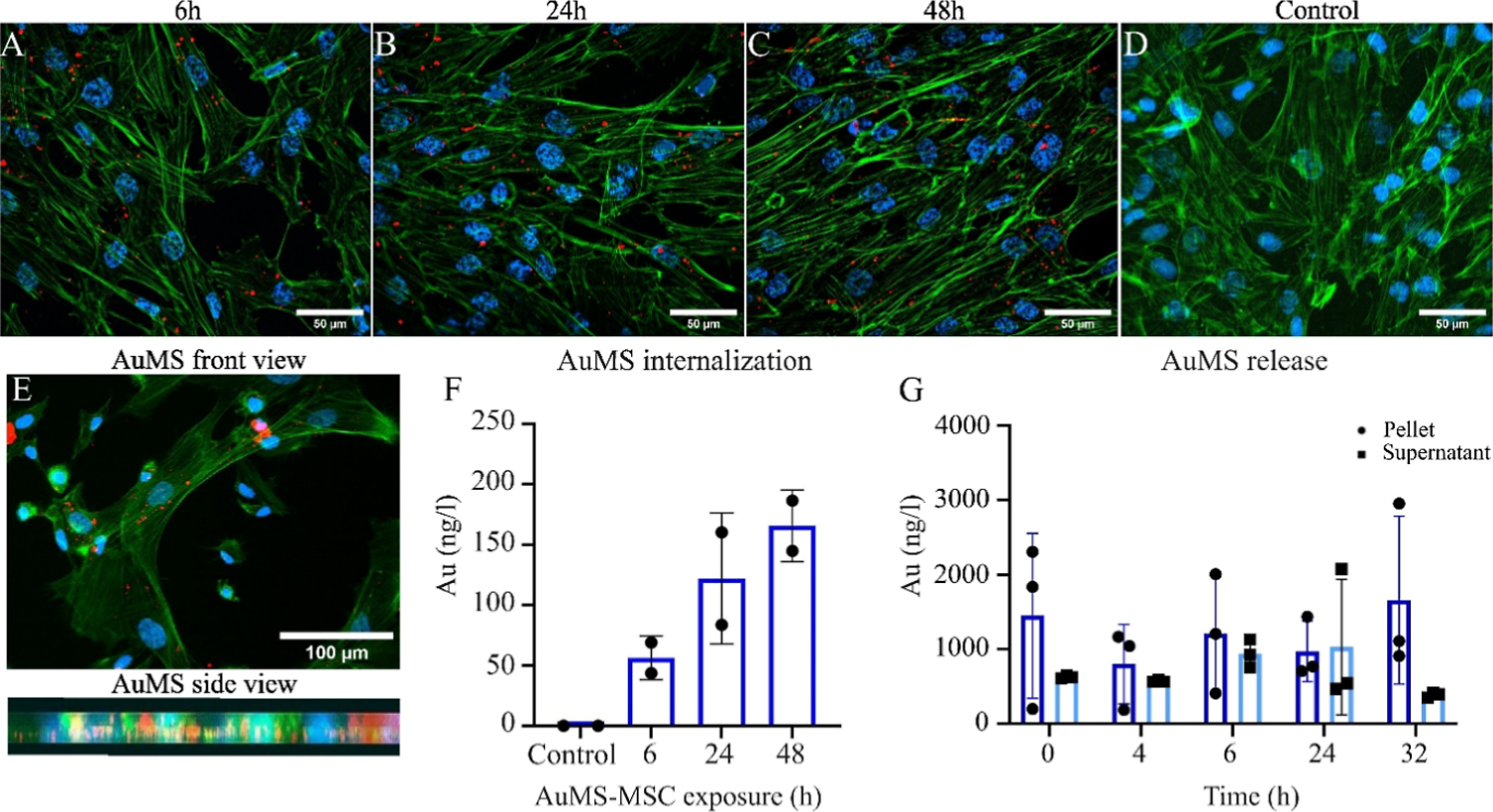
AuMS internalization in MSC over time. MSC were exposed for 6, 24, and 48 h to AuMS in vitro. (A–D) Localization of AuMS (orange) in hMSC, 60× oil objective (blue = nuclei, green = actin filaments) (Scale bars = 50 μm). (E) Front view and side view of AuMS internalized in MSC (Scale bar = 100 μm). (F) Quantifying AuMS internalization in MSC (Au (ng/l)) over time. (G) AuMS release from h-MSC 24 h postexposure (*t* = 0) over time.

To determine AuMS intracellular distribution, MSC were exposed to 50 μg/mL AuMS for 6, 24, and 48 h and NP uptake was assessed using fluorescent microscopy and ICP–MS (Figure 2). Fluorescent images showed the presence of AuMS within the cell cytoplasm. AuMS internalization in MSC occurred 6 h after exposure and increased 24 and 48 h after exposure (Figure 2 A–D). In addition, a 3D presentation of MSC exposed to AuMS for 24 h shows that AuMS are internalized in the same *y* plane compared to the cell nuclei (Figure 2 E; Supporting Information Figure S1). Quantitative results obtained by performing ICP–MS confirm these qualitative observations showing increased Au concentrations (ng/l) over time (Figure 2D). These results indicate that a minimum of 24 h of labeling is required for optimal AuMS internalization. An average concentration of 0.574 ng/L gold was internalized in MSC 24 h postlabeling. Subsequently, to assess AuMS release from MSCs we measured Au concentrations in AuMS-MSC pellets and supernatant 24 h post-AuMS exposure (*t* = 0) (Figure 2G). Our data shows that AuMS remain in the MSC up to 32 h postlabeling.

### 3.3 AuMS Labeling Does Not Alter the Survival, Metabolic Activity, Differentiation, and Immune Phenotype of MSC

**Figure 3:**
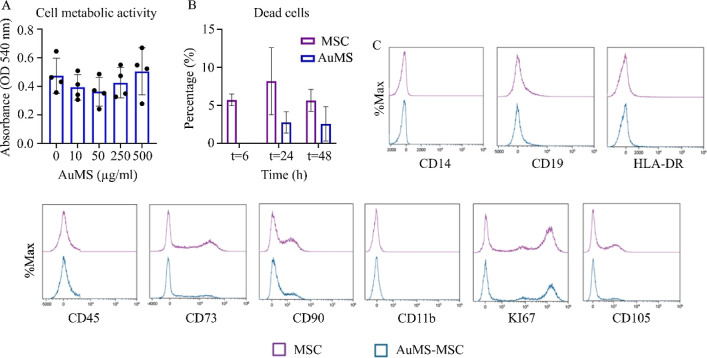
Survival, metabolic activity, and immune phenotype of AuMS-MSC. (A) MSC were treated with 10, 50, 250, and 500 μg/mL AuMS for 24 h, and metabolic activity was measured. 50 μg/mL AuMS represents the concentration that was used for in vivo studies. Three technical replicates were used. Paired *t*-test used to assess differences between each group; *p < 0.05, **p < 0.01, ***p < 0.001, ****p < 0.0001. (B) Percentage dead cells 6, 24, and 48 h post-AuMS exposure (C) Representative histograms of 3 technical replicates. MSC specific markers (CD73, CD105 and CD90) and immune cell lineage markers (CD11b, CD14, CD19, CD45, HLA-DR) were tested. Purple lines represent MSC and blue lines represent AuMS-MSC.

After the successful synthesis and labeling of MSC with AuMS, the influence of NP labeling on MSC survival and metabolism was assessed by live/dead and MTT assay. MSC metabolic activity was measured 24 h after AuMS exposure to dosages ranging from 10 to 500 μg/mL. No significant inhibition of MSC metabolic activity was observed after exposure to AuMS compared to unlabeled MSC (Figure 3A). A live/dead staining on nonfixed MSC was performed 6, 24, and 48 h after MSC exposure to 50 μg/mL AuMS. No differences in the ratio live/dead MSC were observed when comparing MSC exposed to AuMS and control (nonlabeled MSCs) over time (Figure 3B; Supporting Information Figure S2).

To investigate the potential impact of AuMS labeling on MSC phenotype, we analyzed surface marker expression of MSC after 24 h exposure to 50 μg/mL AuMS using flow cytometry. Our results showed that AuMS-MSC retained their characteristic immunophenotype as confirmed by the expression of CD73, CD90, and CD105 markers. Similar to unlabeled MSC, AuMS-MSC did not express CD11b, CD14, CD19, CD45, and HLA-DR. Lastly, AuMS labeling did not affect the proliferative abilities of MSC as shown with unaffected KI67 (Figure 3C) expression and cell numbers (Supporting Information Figure S3).

### 3.4 AuMS Labeling Does Not Change the Immunomodulatory Capacity of MSC

**Figure 4:**
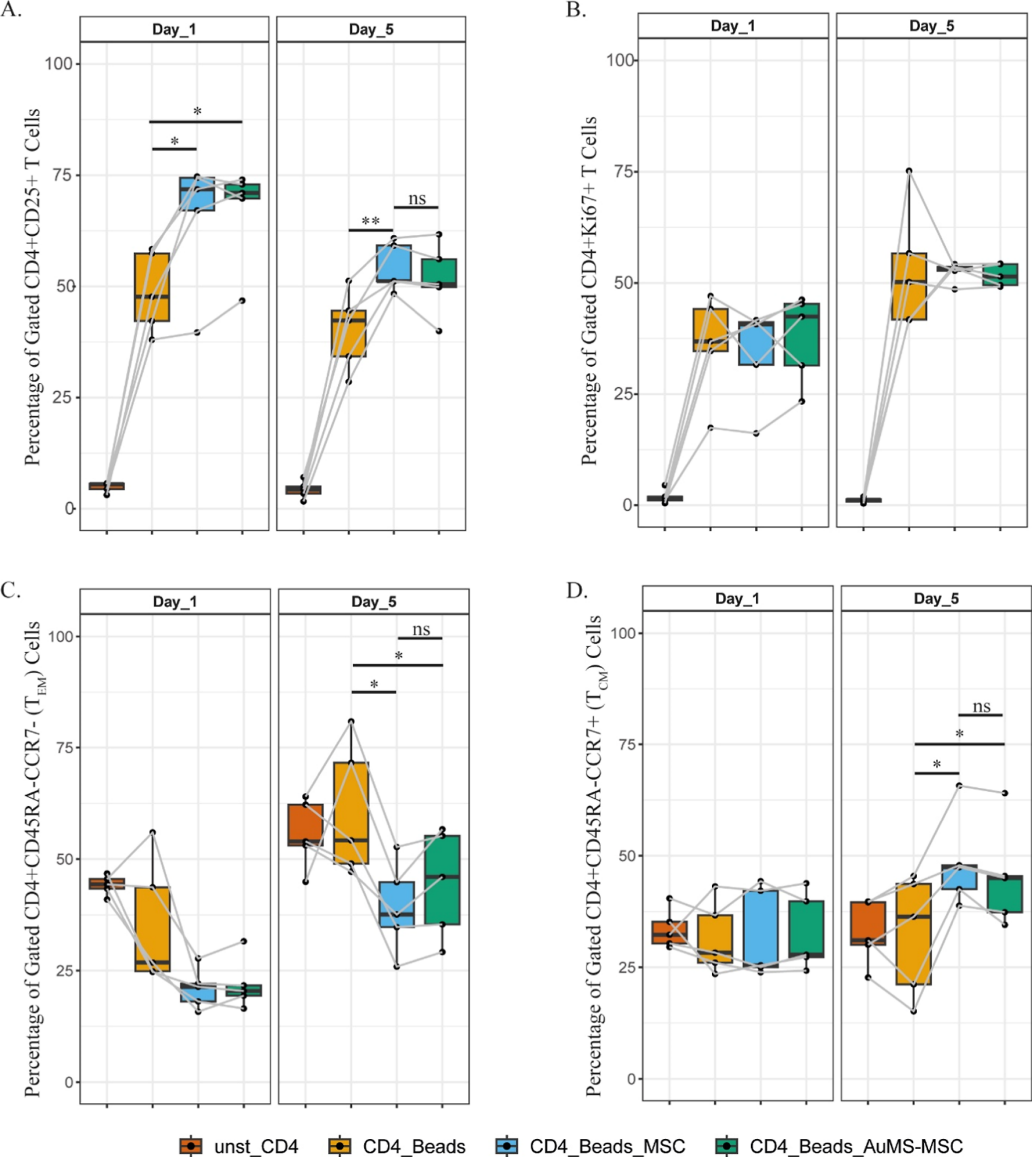
Immunomodulatory effect of AuMS-MSC on CD4^+^ T cells. αCD3/CD28 preactivated CD4^+^ T cells were cocultured with either MSC or AuMS-MSC. After 24 and 120 h of coculture, the immune phenotype of CD4^+^ T cells was assessed. Percentages of different T cells subsets are shown: (A) CD4^+^CD25^+^, (B) CD4^+^KI67^+^, (C) CD4^+^CD45RA^–^CCR7^–^ (T_EM_), (D) CD4^+^CD45RA^–^CCR7^+^ (T_CM_). Four biological replicates were used. Paired *t*-test used to assess differences between each group; *p < 0.05, **p < 0.01, ***p < 0.001, ****p < 0.0001.

Next, the effect of AuMS labeling on the immunomodulatory capacity of MSC on CD4^+^ T cells and monocytes was investigated. For T cells, purified CD4^+^ cells were prestimulated with αCD3/CD28 beads (CD4_Beads) or remained unstimulated (unst_CD4) for 24 h and subsequently were cocultured with either MSC (CD4_Beads_MSC) or AuMS-MSC (CD4_Beads_AuMS-MSC). Stimulation of CD4^+^ T cells with αCD3/CD28 beads increased the expression of CD25, which was further enhanced in the presence of MSC. This enhanced effect of MSC was not affected when MSC were labeled with AuMS (Figure 4A). Considering proliferation, a trend toward a decreased CD4^+^ T cell proliferation was observed in the presence of MSC, which was similar in the presence of AuMS-MSC (Figure 4B). Lastly, the effect of AuMS-MSC on the T cell maturation was investigated in the cocultures. The results showed no difference in the reduction of effector memory (CD4^+^CD45RA^–^CCR7^–^) formation (Figure 4C) and increased central memory (CD4^+^CD45RA^–^CCR7^+^) formation in the presence of either MSC or AuMS-MSC (Figure 4D).

**Figure 5:**
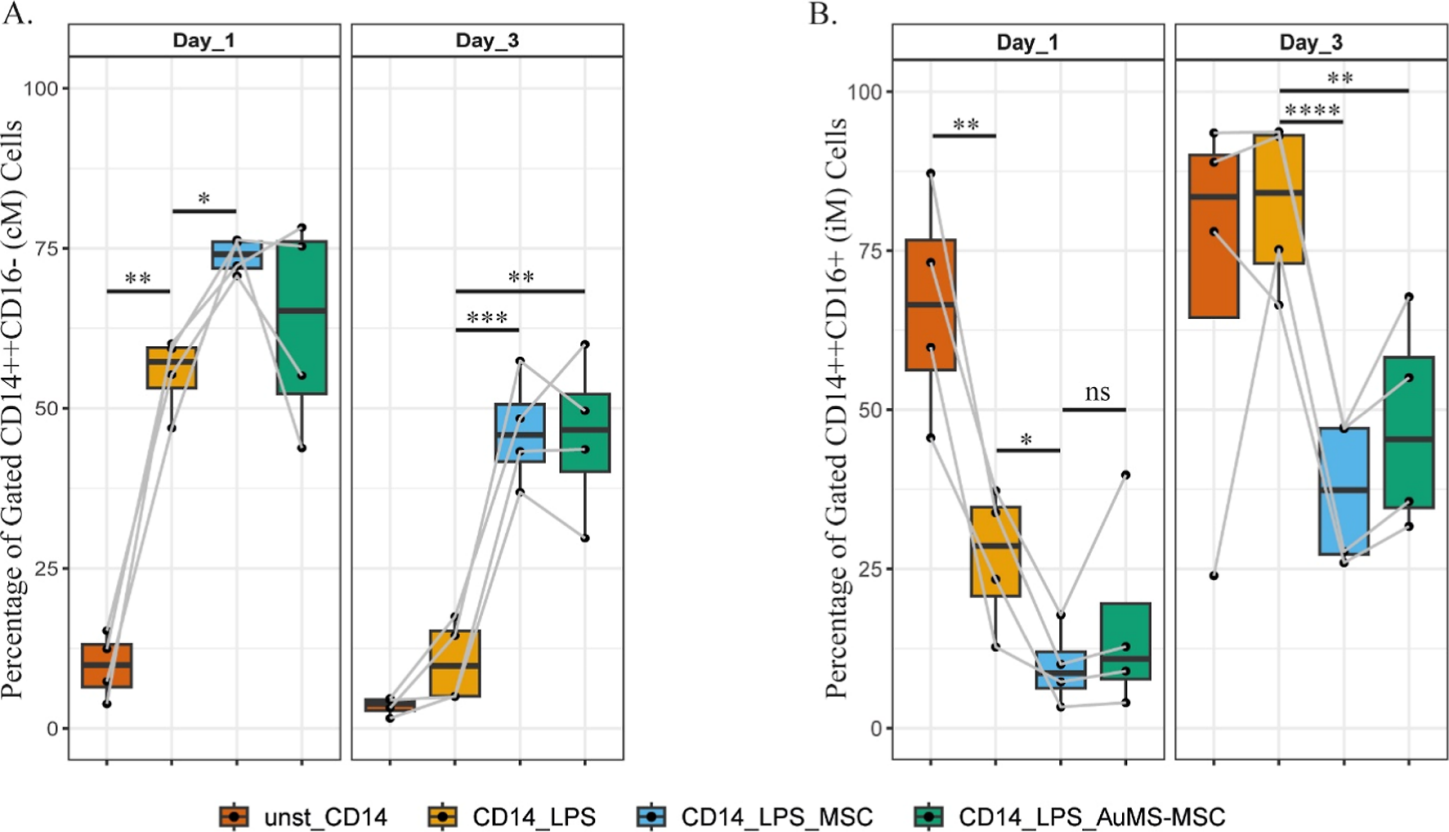
Immunomodulatory effect of AuMS-MSC on CD14^+^ monocytes. LPS preactivated CD14^+^ monocytes were cocultured with either MSC or AuMS-MSC. After 24 and 72 h of coculture, the immune phenotype of CD14^+^ monocytes was assessed. Percentages of (A) CD14^+2^CD16^–^ (cM) and (B) CD14^+2^CD16^+^ (iM) are shown. Four biological replicates were used. Paired *t*-test used to assess differences between each group; *p < 0.05, **p < 0.01, ***p < 0.001, ****p < 0.0001.

Regarding the innate immune response, purified CD14^+^ monocytes were either prestimulated with LPS (LPS, CD14_LPS) or left unstimulated (unst_CD14) for 24 h and cocultured with either MSC (CD14_LPS_MSC) or AuMS-MSC (CD14_LPS_AuMS-MSC) after which monocyte differentiation was measured every other day. LPS stimulation reduced the classical monocyte percentage (Figure 5A) and induced an intermediate phenotype (Figure 5B), while both MSC and AuMS-MSC significantly increased the classical monocyte population on day 3 (Figure 5A). No difference was observed between MSC and AuMS-MSC in regulating monocyte differentiation.

### 3.5 AuMS-MSC can be Detected Both Quantitatively and Qualitatively in a Large Animal Model

**Figure 6:**
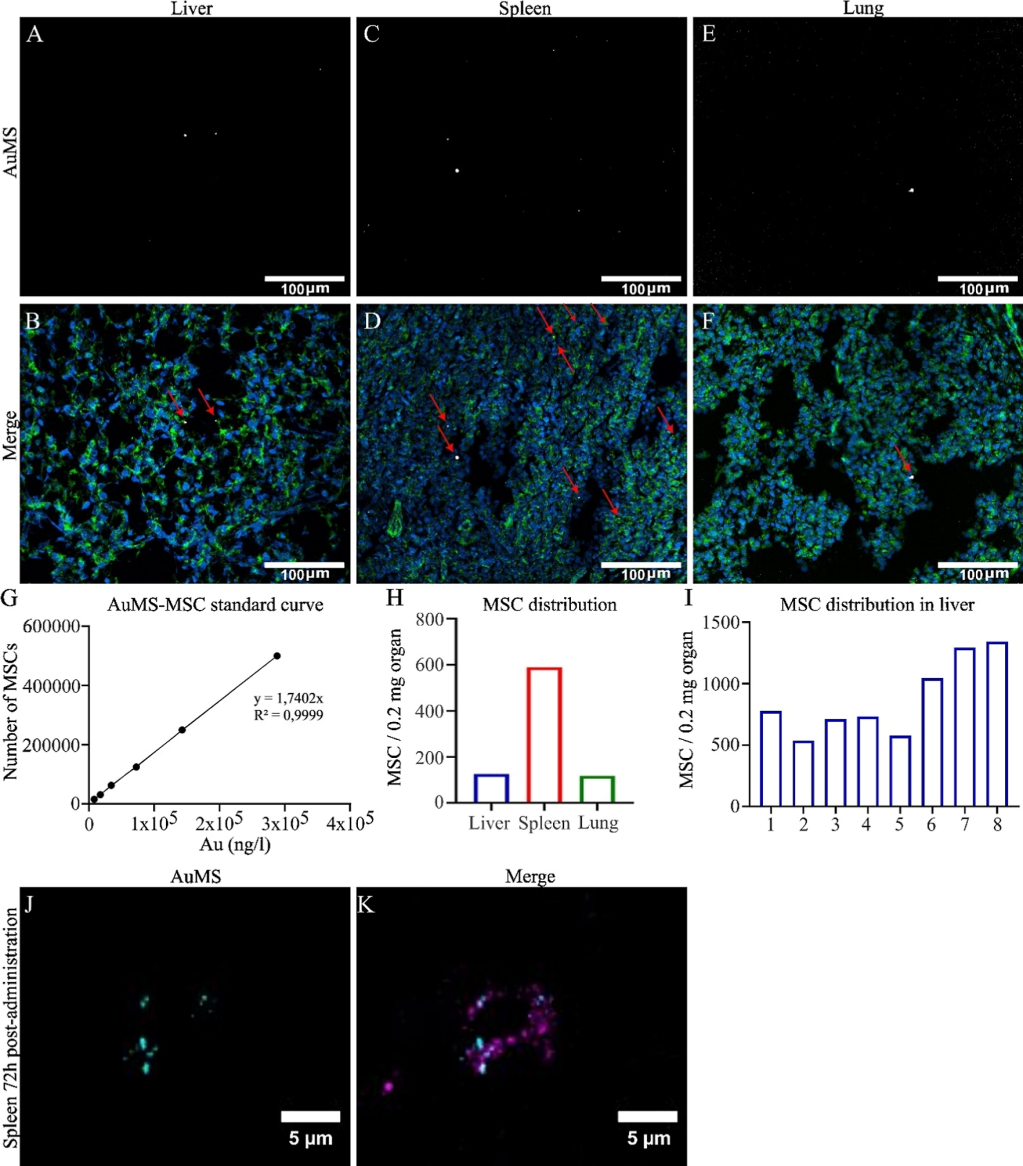
Location and numbers of AuMS-MSC distribution in liver, spleen, and lungs 12 h post-I.V. administration. Lambs received an I.V. dose of 20 M AuMS-MSC/kg body weight immediately after birth. Twelve h after AuMS-MSC administration, lambs were euthanized, and tissues were snap-frozen for cryosectioning and ICP–MS. (A–F) AuMS-MSC positive signal (cyan) was found in lamb liver, spleen, and lung (blue = nuclei, green = actin filaments) (Scale bars = 100 μm). Red arrows indicate the presence of AuMS in the tissue. (G) AuMS-MSC standard curve for the quantification of the total number of MSC in each organ. (H) Quantitative results of MSC distribution in lamb liver, spleen, and lung using ICP–MS. (I) Quantification of MSC distribution in 8 randomly selected liver regions. (J,K) Co-localization between AuMS (Cyan) and human nuclear antigen–antibody (magenta) signal in lamb spleen 72 h after I.V. administration (Scale bars = 5 μm).

After establishing that AuMS internalization in MSC did not affect its metabolic activity and immunomodulatory characteristics, we proceeded with in vivo experiments, as the ultimate goal was to investigate whether our NP could be used as new tools to trace MSC in a large animal model. The safety and biocompatibility of AuMS for cell tracing in vivo were assessed in a preclinical ovine model of preterm birth. Lambs (*N* = 1) were I.V. treated with AuMS-MSC and monitored in the lamb intensive care unit for 12 or 72 h. We observed no differences in breathing frequency, heart rate, diastolic and systolic blood pressure, and blood pH in lambs treated with AuMS-MSC and nonlabeled MSC (Supporting Information Figure S4). Next, as a proof of concept, we determined whether AuMS-MSC was detectable in isolated lamb tissue samples. To this end, AuMS-MSC was assessed quantitatively by elemental analysis and qualitatively by fluorescence microscopy (N = 1). Using fluorescence imaging, our data showed that AuMS-MSC could be localized in lamb liver, spleen, and lung tissue samples. From these organs, the highest fluorescent signal was observed in the spleen (Figure 6A). To quantify the number of MSC per organ, a standard curve of AuMS-MSC was made based on a dilution series from known AuMS-MSC numbers administered to the animal (Figure 6B). Our qualitative data is consistent with our quantitative data showing 5888 MSC/0.2 mg, 1249 MSC/0.2 mg, and 1171 MSC/0.2 mg present in respectively the spleen, liver, and lungs 12 h post-I.V.administration (Figure 6C). To assess whether MSC were homogeneously distributed in the liver we assessed eight biopsies of randomly selected liver regions. Here we show a variation in MSC distribution ranging from 539 MSC/0.2 mg to 1342 MSC/0.2 mg (Figure 6D). Lastly, to confirm that the AuMS positive signal observed in lamb organs correlates to the administered MSC, we performed immunohistochemical staining with a human nuclear antigen–antibody. Co-localization between MSC and AuMS was observed up to 72 h postadministration (*N* = 1), indicating that AuMS were still present in the MSC (Figure 6E). This indicates that AuMS elemental analysis and RITC fluorescence properties are reliable tools for the quantitative and qualitative assessment of MSC biodistribution in large animals 72 h postadministration.

## 4 Discussion

Preclinical research demonstrated the promising therapeutic potential of MSC as a treatment for a large variety of diseases including (multi) organ injury in neonates.^9,43^ To successfully implement MSC therapy in clinical settings, it is crucial to assess several key factors after administration to optimize treatment regimens and improve our understanding of MSC therapy mechanisms.^12^ These factors include biodistribution, dosing, timing, route of administration, functional changes, and the sustainability of MSC. Hence, in this work, we provide a multimodal AuMS-based labeling method that enables qualitative and quantitative assessment of stem cell distribution in a preclinical ovine model of preterm birth. Specifically, the developed NP could be detected via the gold core using ICP–MS analysis to determine cell numbers and had high far-red fluorescent properties to unravel the location of the cells in tissue slices postadministration.

NP’s individual size, surface chemistry, concentration, and shape are key factors that affect their biocompatibility.^44^ Since MSC recognizes the AuMS outer layer which consists of the MS coat it is of significant importance to assess the biocompatibility of the NP shell. Here we showed that AuMS did not affect the metabolic activity of MSC and could be readily internalized. This corresponds with Chen et al. (2019) demonstrating that silica-iron oxide nanoparticle labeling of h-MSCs did not affect cell metabolic activity, indicating an excellent biocompatibility of these nanoparticles.^45^ Besides, this corresponds with prior research that demonstrated no cytotoxicity of MSC 2 h after treatment with RITC-MSN. In addition, they showed that RITC-MSN treatment did not affect MSC proliferation.^46^ Also in a study published by Foglia et al. (2017), it was demonstrated that CaCo-2 cells treated with MS-coated NP at concentrations ranging from 10 μg/mL to 100 μg/mL showed similar cellular viability and proliferation compared to the untreated cells for up to 7 days.^47^ This is in line with our findings, which show that AuMS labeling does not affect MSC proliferation (KI67) (Figure 3C; Supporting Information Figure S3). The United States Food and Drug Administration (FDA) considers silica as “Generally Recognized as safe”. On top of that, a stage I human clinical trial using silica NP (Cornell dots) for molecular imaging has been approved by the FDA.^48^ Next to the excellent biocompatibility of our MS coat, it enables us to load fluorescent dyes which enhances the imaging capabilities of our nanoprobes. Based on their fluorescent properties and elemental analysis, we demonstrated that AuMS cellular uptake occurs rapidly by MSC within 6 h. Moreover, we showed that AuMS remained internalized in the MSC cell cytoplasm for up to 72 h in vitro. This is consistent with prior research showing rapid uptake of silica-coated NP in CaCo-2 cells starting immediately after cell exposure and reaching a peak within 24 h.^47^ AuMS is internalized in MSC for up to 21 days to one month after administration.^34,35^ Our study and other research thus promote AuMS as a biocompatible nanoprobe for quantitative MSC tracing studies for up to 72 h post-I.V. administration. After confirming the biocompatibility of AuMS, the effect of labeling on MSC phenotype and immunomodulatory function was investigated. Previous studies showed that AuMS labeling maintained MSC phenotype.^33,49^ In correlation with the literature, we confirmed that AuMS labeling did not alter the MSC phenotype. Furthermore, since immunomodulation is a hallmark characteristic of MSC and is considered an invaluable tool in supporting the recovery from diseases of prematurity, it is of significant interest to assess the effect of labeling on MSC function.^3,13^ Therefore, we assessed the immunomodulatory capabilities of AuMS-MSC in vitro coculture models using preactivated CD4^+^T and CD14^+^ monocytes. Previously, we showed that MSC can modulate T cell activation, maturation, and proliferation. Especially, a pronounced induction of an anti-inflammatory T cell phenotype with more central memory T cells was observed.^40^ Labeling of MSC with AuMS did not affect these immunomodulatory effects of MSC on T cells. As previously described, MSC are able to skew monocytes from an intermediate to a classical phenotype.^50^ High levels of the intermediate phenotype positively correlate with the LPS-induced sepsis phenotype.^51^ Therefore, inducing the classical monocyte population by MSC exerts a therapeutic effect on monocytes. We show that the capability of MSC to induce an intermediate monocyte population is not influenced by AuMS labeling as they exhibited the same immunomodulatory behavior. This is in agreement with Chen et al. (2019) showing that silica-iron oxide nanoparticle-labeling of h-MSCs did not affect cell differentiation and MSCs preserved their osteogenic and adaptogenic functionalities.^45^

We observed no adverse effects nor abnormalities in preterm-born lamb’s vital parameters (Supporting Information Figure 4) 12 h post-I.V. treatment with AuMS-MSC. This is in accordance with a previous mice study showing no abnormalities in hematoxylin and eosin staining of heart, liver, kidney, and spleen tissue 21 days after treatment with AuMS-MSC. Moreover, it was shown that AuMS-labeled MSC maintain their functional characteristics in vivo.^34^ Since our goal is to unravel the biodistribution, fate, and mode of action of MSC in in vivo models of preterm birth it is highly important to develop nanoprobes that are nontoxic and do not interfere with the characteristics of the assessed treatment strategy.

We demonstrated that our proposed AuMS-based nanoprobes are appropriate for cell labeling to investigate MSC distribution in a large animal model. By labeling MSC with AuMS, we were able to quantify cell distribution based on elemental analysis 12 h post-I.V. administration. Moreover, the exact location of our AuMS-MSC could be investigated by fluorescent analysis of tissue slides. Several studies already demonstrated the successful use of gold nanoprobes for cell tracing studies in mice. For example, noninvasive tracking of monocytes in atherosclerotic plaques and stem cell tracking for Duchenne muscular dystrophy using CT.^52,53^ CT is a commonly used technique for the detection of AuMS as a result of the high X-ray absorption of gold.^34,35,53^ Although CT is considered a quantitative cell tracing technique, obtaining quantitative data from CT images is still difficult.^54,55^ CT has low sensitivity and therefore a need for high payloads in cells to create sufficient attenuation for detection.^56^ Our NP’s design enabled us to both quantify and qualify MSC biodistribution based on respectively ICP–MS and fluorescence microscopy. ICP–MS has a lower detection limit that can extend to parts per trillion. ICP–MS’ high sensitivity and low detection limits are of significant importance for the quantification of nanoprobe labeled-MSC tracing on single cell level in large animals. Moreover, qualitative data based on AuMS’ fluorescence provides MSC location-specific information. These features together will expand our understanding of MSC fate, location, and mode of action postadministration, this is crucial knowledge for the successful implementation of MSCs-based therapies in the clinic. To the best of our knowledge, we are the first to investigate AuMS nanoprobes for quantitative cell tracing using ICP–MS in a large animal model. Interestingly, our quantitative and qualitative data both indicate that many I.V. administered AuMS-MSC can be found in the spleen. This finding is in line with previous work showing that aged MSC were only found in the spleen of young recipient mice.^57^ Moreover, Xue et al. (2022) demonstrated the critical role of the spleen in enhancing MSC-induced immunomodulation.^58^

As a proof of concept, we determined whether nanoprobe labeling remains preserved in vivo by assessing the colocalization of a MSC-specific marker and AuMS. Our results showed colocalization between MSC-specific signal and AuMS, indicating labeling stability for at least 72 h postadministration. In addition, we show AuMS signal near HNA positive signal (Supporting Information Figure S5). This is in agreement with prior findings indicating that a large majority of I.V. administered MSC disappear within 3 days postadministration and almost all MSC disappear 7 days postadministration.^59,60^ Hence, our nanoprobes enable us to quantitatively and qualitatively trace MSC and indicate the fate of MSC postadministration in large animals.

## 5 Conclusions

In conclusion, we show that labeling MSC with AuMS does not affect MSC metabolism, phenotype, and immunomodulatory capacity in vitro. As a proof of concept, we show that AuMS are safe stem cell tracers for both qualitative and quantitative assessment of MSC’s biodistribution and fate in large animals. Using AuMS can provide crucial information in the guidance of MSC-based treatment for (multi) organ injury in various disease models toward a more successful clinical translation.

## Supporting Information Available

3D projection AuMS internalization in MSC; live–dead staining of MSCs and AuMS exposed MSCs; MSC vs AuMS-MSC proliferation; list of monoclonal antibodies; lamb vital parameters post-I.V. AuMS-MSC treatment; AuMS internalization in vivo (PDF)
(AVI)

## Supplementary Material

mt4c01714_si_001.pdf

mt4c01714_si_002.avi

## Abbreviations

APTES: 3-aminopropyl triethoxysilane
AuMS: silica-coated gold nanoparticles
AuMS-MSC: AuMS labeled MSC
AuNP: gold nanoparticles
CT: computed tomography
CTAB: cetrimonium bromide
FDA: U.S. Food and Drug Administration
HNA: human nuclear antigenantibody
HPS: human-pooled serum
I.V: intravenously
ICP-MS: inductively coupled plasma mass spectrometry
LPS: lipopolysaccharide
MPTES: 3-mercaptopropyl trimethoxysilane
MRI: magnetic resonance imaging
MS: mesoporous silica
MSC: mesenchymal stem cells
NP: nanoparticles
PBMC: peripheral blood mononuclear cells
RITC: rhodamine B isothiocyanate
SPECT: single photon emission computed tomography
TEOS: tetraethyl orthosilicate
