# Supplementary material for: Gold Mesoporous Silica-Coated Nanoparticles for Quantifying and Qualifying Mesenchymal Stem Cell Distribution; a Proof-of-Concept Study in Large Animals
Source: ACS Appl Bio Mater. 2025 Feb 3;8(2):1511–23. doi: 10.1021/acsabm.4c01714 (PMC11836931; doi:10.1021/acsabm.4c01714)
Supplement: Supplementary file 1 — mt4c01714_si_001.pdf [file mt4c01714_si_001.pdf]

**Supporting Information**

# Gold mesoporous silica-coated nanoparticles for quantifying and qualifying mesenchymal stem cell distribution; a proof-of-concept study in large animals

Lotte C.C. Smeets[1,3,4‡], Ezgi Sengun[2‡], Chloe Trayford[1], Bram van Cranenbroek[2], Marien I. de Jonge[2], Katiuscia Dallaglio[5], Matthias C. Hütten[3,4], Mark Schoberer[6], Daan R.M.G. Ophelders[3,4], Tim G.A.M. Wolfs[3,4], Renate G. van der Molen[2‡] and Sabine van Rijt[1‡*]

[1]MERLN Institute for Technology-Inspired Regenerative Medicine, Maastricht University, P.O. Box 616, 6200 MD Maastricht, The Netherlands

[2]Department of Laboratory Medicine, Laboratory of Medical Immunology, Radboud University Medical Center Nijmegen, 6500 HB Nijmegen, The Netherlands

[3]Department of Pediatrics, Maastricht University Medical Center+, MosaKids Children's Hospital, 6200 MD Maastricht, The Netherlands

[4]GROW Research Institute for Oncology and Reproduction, Maastricht University, 6200 MD Maastricht, The Netherlands

[5]Global Rare Diseases R&D, Chiesi Farmaceutici S.p.A., 43122 Parma, Italy

[6]Division of Neonatology, Department of Pediatrics, University Hospital RWTH Aachen, 52074 Aachen, Germany

‡ Authors contributed equally.

* Corresponding author:

Sabine van Rijt, PhD

Associate Professor

Department of Instructive Biomaterials Engineering

MERLN Institute for Technology-Inspired Regenerative Medicine, Maastricht University

E-mail: s.vanrijt@maastrichtuniversity.nl

Supporting
information for public

**Figure S1. AuMS internalization in MSC.** MSC were cultured in AuMS-containing medium (50 µg/l) for 24h. AuMS (red) is internalized in the MSC cytoplasm (green) near the cell nuclei (blue) (Scalebar =100 µm).

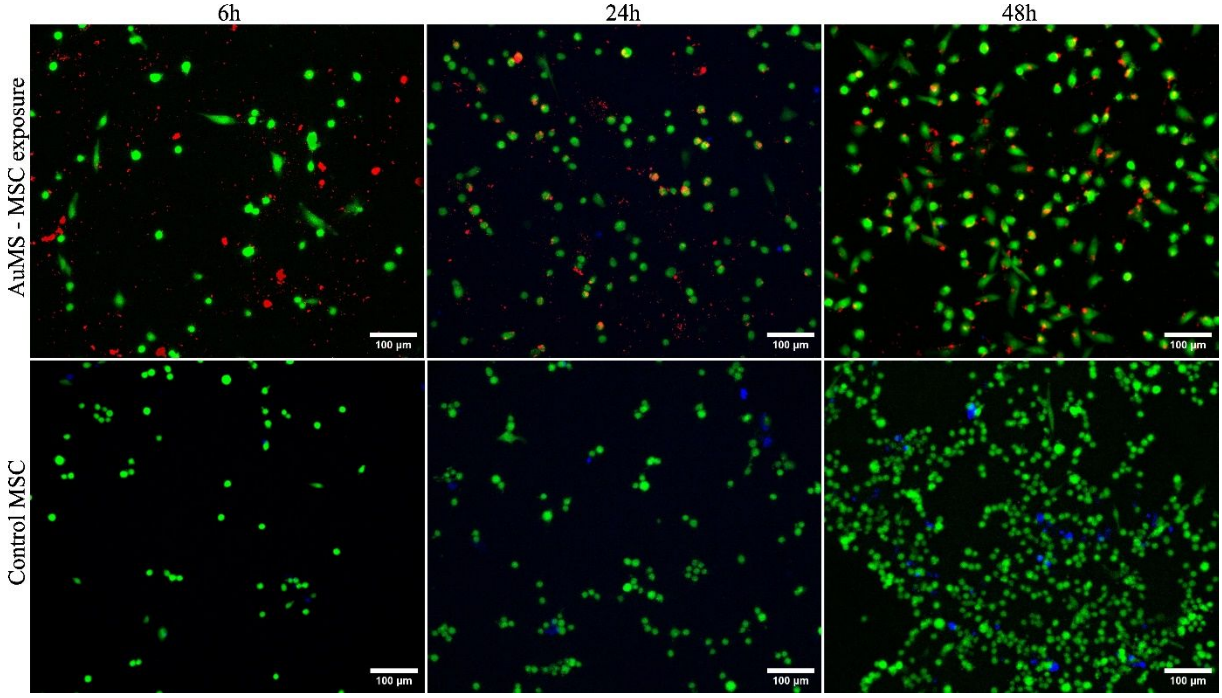


**Figure S2: Live dead staining.** H-MSCs were exposed to AuMS for 6, 24, and 48 h. Live cells (green) and dead cells (blue) AuMS (red) (Scale bars = 100 µm).

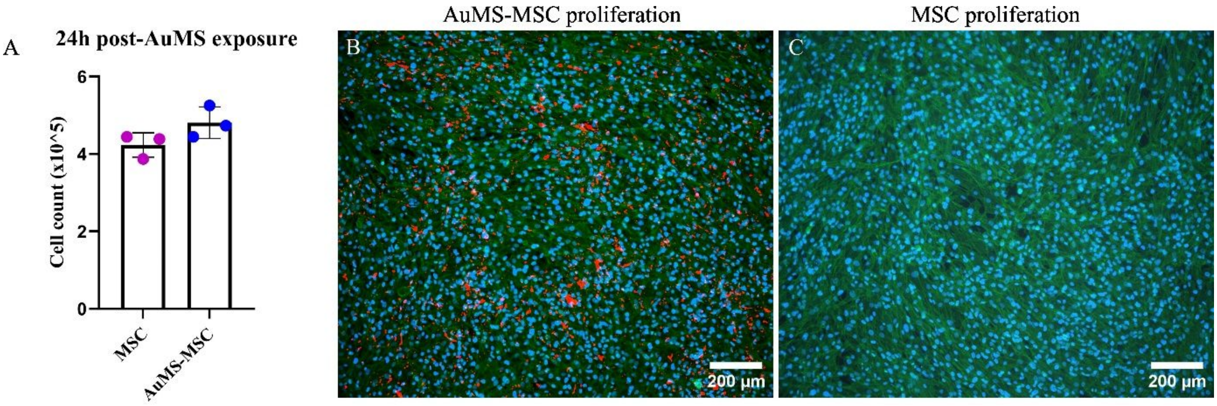


**Figure S3: MSC vs. AuMS-MSC proliferation.** (A) AuMS-MSC (blue) and MSC (purple) proliferation 24 h post-AuMS exposure. (B-C) Representative visualization of AuMS-MSC and

MSC proliferation 24 h post-AuMS exposure. (Blue = nuclei, green = actin filaments, red = AuMS) (Scale bars = 200 µm).

**Table S1.** List of monoclonal antibodies used in three panels.

| Antibody | Producer | Catalog Number |
|---|---|---|
| **CD4 – PE Cy5.5** | Beckman Coulter | B16491 |
| **CD11b – PE Dazzle 594** | BioLegend | 301348 |
| **CD14 – PerCp Cy5.5** | BioLegend | 301824 |
| **CD16 – BV711** | BD Bioscience | 563127 |
| **CD19 – APC AF750** | Beckman Coulter | A94681 |
| **CD25 – PE Cy7** | Beckman Coulter | 557741 |
| **CD45 – Krome Orange** | Beckman Coulter | B36294 |
| **CD45RA – ECD** | Beckman Coulter | B49193 |
| **CD73 – APC** | eBioscience | 25-1057-42 |
| **CD90 – BV711** | BioLegend | 328140 |
| **CD105 – PE Cy7** | eBioscience | 25-1057-42 |
| **HLA-DR – Pacific Blue** | Beckman Coulter | B36291 |
| **KI67 – AF488** | BD Bioscience | 561165 |
| **KI67 – AF647** | BD Bioscience | 558615 |

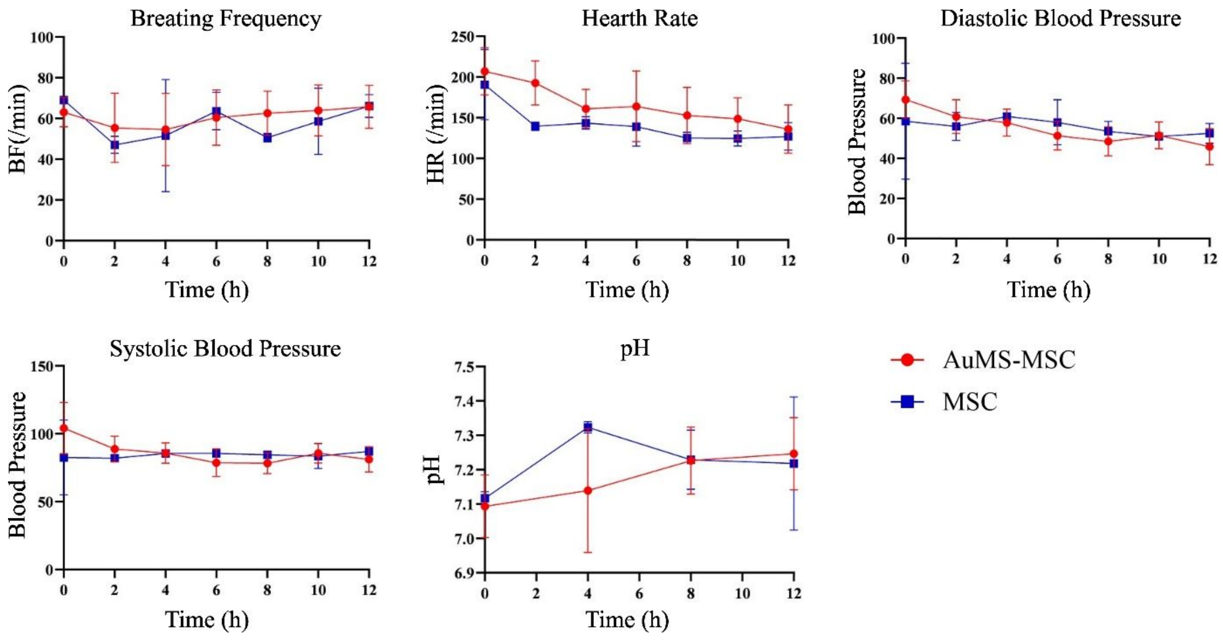

**Figure S4. Lamb's vital parameters during their first 12 h at the lamb intensive care unit.** Lambs received AuMS-MSC (red) or MSC (blue) immediately after preterm birth. Vital parameters were monitored every 2 h for 12 h.

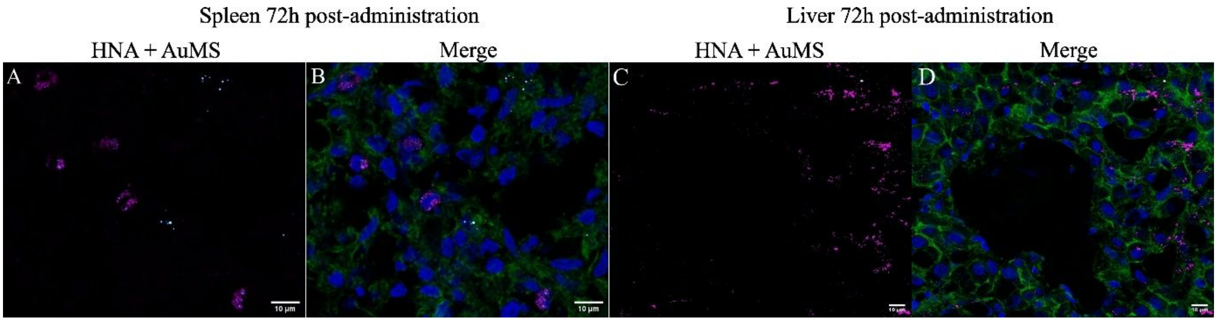


**Figure S5. AuMS internalization in the spleen of preterm born lambs 72 h post-administration.** (A, C) AuMS (cyan) and HNA (magenta) positive signal. (B, D) Merge picture of spleen and liver histology nuclei (blue), actin filaments actin filaments (green)**,** AuMS (cyan), and MSC (magenta) (Scale bar = 10 µm).
